# Plant and soil nutrient stoichiometry along primary ecological successions: Is there any link?

**DOI:** 10.1371/journal.pone.0182569

**Published:** 2017-08-07

**Authors:** Francesca Di Palo, Dario A. Fornara

**Affiliations:** 1 School of Geography and Environmental Sciences, University of Ulster, Coleraine, United Kingdom; 2 Agri-Food and Biosciences Institute (AFBI), Newforge Lane, Belfast, United Kingdom; Stellenbosch University, SOUTH AFRICA

## Abstract

Ecological stoichiometry suggests that plant Nitrogen (N)-to-Phosphorus (P) ratios respond to changes in both soil N:P stoichiometry and soil N and P availability. Thus we would expect that soil and plant N:P ratios be significantly related along natural gradients of soil development such as those associated with primary ecological successions. Here we explicitly search for linkages between plant and soil N:P stoichiometry along four primary successions distributed across Europe. We measured N and P content in soils and plant compartments (leaf, stem and root) of 72 wild plant species distributed along two sand dune and two glacier successions where soil age ranges from few to thousand years old. Overall we found that soil N:P ratios strongly increased along successional stages, however, plant N:P ratios were neither related to soil N:P stoichiometry nor to changes in soil N and P availability. Instead changes in plant nutrient stoichiometry were “driven” by plant-functional-group identity. Not only N:P ratios differed between legumes, grasses and forbs but each of these plant functional groups maintained N:P ratios relatively constant across pioneer, middle and advanced successional stages. Our evidence is that soil nutrient stoichiometry may not be a good predictor of changes in plant N:P stoichiometry along natural primary ecological successions, which have not reached yet a retrogressive stage. This could be because wild-plants rely on mechanisms of internal nutrient regulation, which make them less dependent to changes in soil nutrient availability under unpredictable environmental conditions. Further studies need to clarify what underlying evolutionary and eco-physiological mechanisms determine changes in nutrient stoichiometry in plant species distributed across natural environmental gradients.

## Introduction

A long-standing question in plant ecology remains about what underlying eco-physiological mechanisms control plant species occurrence and distribution along environmental gradients. Numerous studies over the last century suggest that the answer partly lies within the concept of “niche” whereby niche differences in plant-resource-use and plant competitive abilities ultimately influence the composition of plant assemblages under specific environmental conditions [1–5]. Other studies suggest that a better mechanistic understanding of how plant species adapt and establish along environmental gradients should be sought at the organismic or cellular level, for example, by addressing physiological and genetic features among coexisting plant species [6]. This could be seen as a ‘reductionist’ approach (compared to community-level investigations) and has been recently ‘reinforced’ by biological stoichiometric theory [7], which suggests that element ratios of individual plant species (e.g., N:P ratios) and of different plant compartments might reflect changes in environmental conditions. The idea is that variation in plant growth rates can be strongly related to changes in soil N and P availability and to the degree of N:P co-limitation under specific environmental conditions [8–10].

Previous studies show how variation in soil N:P ratios can influence plant biomass and plant nutrient concentrations [10–12] and also how changes in soil N:P ratios can be more important than absolute changes in total soil N and P content when explaining variation in plant stoichiometry [13–14]. Similarly, changes in plant N:P ratios not only can be related to changes in soil nutrient availability [15] but can also be related to the degree of nutrient limitation experienced by coexisting plant species [16].

Here we test the idea that changes in N:P stoichiometry of herbaceous plant species may reflect either changes (1) in soil N:P stoichiometry or (2) in soil N and P availability along natural gradients of soil development. Although patterns of plant species occurrence along environmental gradients may have an underlying stoichiometric explanation [7], few studies have addressed whether plant and soil nutrient stoichiometry may be related along primary ecological successions.

We take a stoichiometric approach and measure N:P ratios of different plant above- and belowground compartments asking whether these nutrient ratios may change as a response to changes in soil nutrient stoichiometry and soil nutrient availability. We selected two sand-dune and two pro-glacial systems across Europe, which represent classic examples of primary ecological successions, where soils have evolved in sequence at successively later times and in parallel with increasingly older plant communities [17]. Our successions include soil substrates, which have developed within a time-span of 0–1000 years and it is unlikely that they have reached a retrogressive stage whereby available soil P has become particularly limiting for plant growth [18]. Nevertheless, glacier and sand-dune ecosystems offer a unique opportunity to address how changes in soil nutrient availability may be reflected in plant growth [19] and whether variations in soil N:P ratios along increasingly older stages of the primary successions could ultimately influence plant N:P stoichiometry.

In our study we explicitly seek potential relationships between plant and soil N:P stoichiometry across each of three well defined successional stages (i.e. early, middle and advanced) and ask whether plant N:P ratios could predictably change across the soil development gradient. We expect plant growth on young soils be more limited by N than by P [20–21], which should lead to lower plant N:P ratios when compared to plant N:P ratios of older soils. A recent nutrient fertilization experiment [22] shows that changes in soil fertility (e.g., N and P availability) are important, because these nutrient concentrations tends to shift in a systematic way until it is possible to separate plants that grow under different fertility conditions. Important aspects to consider, however, is whether changes in plant stoichiometry reflect changes in soil nutrient concentrations under more natural conditions or whether plant stoichiometry is more ‘controlled’ by evolutionary history traits and/or by functional trait differences between co-existing plants [9]. For example plant functional group identity (e.g., grasses vs. legumes vs. forbs) can play an important role in influencing plant resource use and acquisition as well as plant biomass production [23–25]. Our study aims to test the following predictions [see 9]:

1. If plant N:P stoichiometry is largely determined by changes in soil N:P ratios or in N and P availability along successional stages (e.g. early vs. advanced stages), we would expect soil and plant N:P ratios be positively related along a gradient of soil development.
2. If plant N:P stoichiometry is more a reflection of plant functional identity (e.g., legumes vs. grasses), we would expect no relationships between soil and plant stoichiometry along successional stages, but we would expect differences in N:P ratios among plant species growing within the same successional stage.

## Material and methods

### Study sites

We selected four study sites across Europe (S1 Fig), each being representative of a natural primary ecological succession. We specifically focused on two sand dunes and two pro-glacier systems, which are distributed across very different environmental and climatic conditions. The first sand dune succession, Praia Carrapateira, is located within the Parque Natural do Sudoeste Alentejano e Costa Vicentina (PNSAVC) in the Algarve, Portugal (37°36'N, 8°40'W); the second sand dune system is located within the Umbra Natural Reserve, Northern Ireland, UK (55° 9'N, 6° 51'W). The sand dune systems include well-drained alkaline sandy soil with an average pH of 8.0. These soils are characterized by a prevalent calcareous component, derived from limestone parent rock. The two glacier primary successions are located at the foot of the Monte Rosa Massif in the Anzasca Valley, Northern Italy and have been influenced by advances and retreats of two glaciers, the Belvedere Glacier (45° 96’N, 7° 92’E) and the Locce Glacier (45° 95'N, 7° 92'E). The two glacier systems include acidic silt-sandy soils, whose parent rock of gneiss determines the relatively low soil pH = 5. Average climate data ranging 1950–2000 (WorldClim-Global Climate Database, www.worldclim.org), show mean temperatures of 15.9°C for Praia Carrapateira, 8.5°C for Umbra Reserve and 5°C for the Anzasca Valley Glaciers. Annual mean precipitation values are 563 mm for Carrapateira, 1065 mm for Umbra and 1695 mm for the Monte Rosa Eastern Face, here mainly represented by snow precipitation (S1 Table). Permission to access the Umbra-Magilligan Dune System within the Designed Special Area of Conservation (SAC; Northern Ireland) was issued by Ulster Wildlife Trust. Permission to access the Praia Carrapateira dune system was issued by the Parque Natural do Sudoeste Alentejano e Costa Vicentina (PNSAVC; Portugal). Macugnaga city council allowed access to the non-protected areas adjacent to the Alpine glaciers in the upper Anzasca Valley (Italy). The field studies did not involve endangered or protected species.

#### Geomorphology and plant community composition

The four vegetation successions have been influenced by climatic events that mainly occurred during the Holocene. Both dune systems appear particularly shaped by the Atlantic transgression and regression due to glacial and interglacial cycles and during the more recent Little Ice Age (16^th^-19^th^ centuries). Fluctuations of the alpine glaciers during this period were responsible for setting the composition and structure of vegetation on stabilized moraines (middle stage) within the primary successions analysed in our study [26]. The Umbra Reserve succession, included in the Magilligan Dune System Special Area of Conservation (SAC), is characterized by the presence of embryonic shifting dunes, which are rare because they cover less than 1000 hectares across all UK. These dunes are dominated by the *Ammophila arenaria*. More stabilized dunes (i.e. at intermediate and later stages) include common species such as *Festuca rubra*, *Galium verum*, *Helictotrichon pubescens* and *Thymus polytrichus*. These dunes started developing between 5,000–2,000 years BP and radiocarbon dating suggests that they formed during mid-and late Holocene [27]. The Praia Carrapateira dune system in South West Portugal is one of the best-preserved littoral systems of Europe, which hosts endemic plant species. Here the foredunes are colonized by early stage plants (i.e. *Ammophila arenaria* and *Lotus creticus*) followed by more stable grassland communities dominated by *Lagurus ovatus* and by *Balduina angustifolia*. Although there is little information on the dune systems of south-western Portugal, the active dune-fields were formed during the Holocene after stabilization of the sea level [28]. According to these authors, periods of more active dune building during the late Holocene have occurred, probably between 15^th^ and 19^th^ centuries. We attribute our mid-successional stages to a series of geomorphological events associated with changes in climatic conditions during the Little Ice Age and changes in land use [28]. The early stage is included in the recent dune building episodes identified in Portugal (1770–1905 AD) [29] and still subject to remodelling. At our Alpine sites early plant communities started developing on moraine substrates recently abandoned by the Belvedere Glacier and include pioneer species such as *Linaria alpina* and *Oxyria digyna*. Mid-successional communities on more stabilized terrains, mainly ice-free since the 19^th^ century include common species such as *Poa alpina*, *Trifolium pallescens*, *Lotus alpinus* and *Achillea moscata*. Finally advanced stages, undisturbed by glaciers during the Holocene, include species-rich communities of *Phleum alpinum*, *Geum montanum* and *Trifolium alpinum* [30]. Previous geomorphological studies suggest that our pioneer and middle-stage plant communities started developing on moraine deposits formed during 15^th^ and 20^th^ centuries [26, 31–32]. Plant community changes are similar along the Locce Glacier where pioneer species include *Agrostis schraderiana*, *Poa alpina* and *Leucanthemopsis alpina*, whereas a more stabilized vegetation also includes *Festuca halleri*, *Lotus alpinus* and *Rumex scutatus*. A reconstruction of the historical evolution of the vegetation succession and soil development in the area surrounding the Locce Glacier has to be referred to the same serial of events that characterized the Belvedere Glacier (S1 Table).

### Experimental design

In each of the four primary successions we identified three successional stages (early, middle and advanced). Within each successional stage we randomly selected 6 sampling sites (at least 10 meters apart from each other) where we collected plant and soil samples (S2 Fig). Our aim was to select plant species not based on their biomass contribution to the plant community (i.e. dominance) but based on individuals’ recurrence (i.e. frequency). We chose to use frequency instead of dominance in order to avoid monotype-like inferences and to select the most-representative species for each successional stage and soil substrate age. An open reel measuring tape was stretched across each sampling site for a length of 10 meters and all flourishing plant species along the transect were recorded on a datasheet including information on their functional identity (i.e. legume, grass, forb). The transect method was repeated 6 times within each successional stage (S2, S3, S4 and S5 Tables).

#### Plant sampling and laboratory analysis

Our aim was to collect individuals of different plant species, which are representative of three major plant functional groups: legumes, grasses and forbs. The ecological role of these functional groups within grassland ecosystems has been addressed in previous studies [33–34]. Entire plant samples were collected between April and August 2011, which included the peak-growing season for most of the plant species selected. Available plant species from each plant functional group were identified and collected *in situ* within each successional stage. Some species were recurrently found in the same stage (Early, Middle or Advanced) of two different successions (e.g. *Trifolium alpinum* was found in the advanced stage of both Belvedere and Locce Glaciers) (S6 Table). Other species were found in two different successional stages of the same succession (e.g., *Plantago lanceolata* was found in Middle and Advanced stages of the Umbra sand dune system) (S6 Table). We then randomly collected ten individuals of each representative plant species at one of the six sampling sites established within each successional stage (2 representative species × 3 functional groups × 3 successional stages × 4 primary successions = 72 data points). Plant material was stored under a portable plant press to absorb moisture. Individuals did not show any sign of herbivory and in general grazing disturbance by wild animals in these protected areas is considered low. We then sorted plant biomass into leaves, stems and roots. Roots were gently washed over a steel mesh (1 mm size) to remove soil and any organic detritus. All dry plant material was ground to powder using a Mixer Mill MM 200 (RETSCH, Verder Group, Germany). We analysed leaves, shoots and roots for total P (%) and total N (%) content. Total N (%) was measured by combustion and gas chromatography using a COSTECH Analytical Element Combustion System 4010 (ESC 4010, Costech Analytical Technologies, Inc., Valencia, CA). Total P (%) was measured by flow injection using a Lachat’s QuickChem 8500 Series 2 (Lachat Instruments, Hach Company, Loveland, US).

#### Soil sampling and laboratory analysis

Soil samples were collected in April-May 2011 at Praia Carrapateira, in June 2011 at Umbra and in August 2011 at the two Alpine sites. Soils were taken between 0 and 20 cm depth, using a 5 cm diameter soil metal corer. For each of the plant species selected across the three successional stages, soil cores of 393 cm^3^, were collected each beside three of the ten individuals harvested and mixed before storing them in sealing bags at 5°C. The 72 soil samples were sieved, through a 2 mm mesh size to remove roots and gravel, and then dried at 60°C for 3 days. Before oven-drying the soil samples, 20 grams of fresh soil subsamples were extracted with 1*M* KCl solution, shaken for 30 minutes, settled overnight at 4°C, and analysed for ammonium (NH_4_^+^) and nitrate (NO_3_) concentrations by ion chromatography using a Lachat QuickChem 8500 (Lachat Instruments, Hach Company, Loveland, Colorado, USA). Another set of soil sub-samples were incubated for a month in the dark with sufficient water added twice to keep constant moisture at 22°C. After 30 days samples were extracted with 1*M* KCl solution and analysed for NH_4_^+^- N and NO_3_^-^ - N as previously. Initial values of NH_4_^+^ and NO_3_^-^ were subtracted from final NH_4_^+^ and NO_3_^-^ concentrations of the incubated samples to calculate potential net soil N mineralization rates. Total soil N (%) was measured by combustion and gas chromatography using a COSTECH Analytical ECS 4010 instrument (Costech, Valencia, California). Soil P available for plant uptake was measured using a water extraction empirical soil-test performed on air-dried soil subsamples. Previous studies show that water-extraction analysis is a reliable indicator of available P across a wide-range of soil types [35–37]. A recent study [38], shows that current soil P extraction methods, such as Olsen and Melich 1 and 3, are not adequate for soils with high pH as our sand dune soils, which have pH = 8. Water-extractable P is not dependent on soil type and provides a good indication of plant-available P [37]. Sodium hydroxide (NaOH) fusion was applied to air dried soil to measure total soil P (%) using a spectrophotometer set with deionised water.

#### Data analysis

We tested (1) whether and how soil and plant N:P stoichiometry would change across successional stages, (2) whether soil and plant N:P stoichiometry were significantly related along increasingly older stages of four primary successions, and (3) whether changes in plant N:P ratios were associated with plant functional group identity (i.e. legumes, grass and forbs). Because we found that soil N:P ratios significantly increased across successional stages [39], we treated as covariate levels some soil chemical and stoichiometric parameters (i.e. soil N:P, net N mineralization rates, available P, total soil N and total soil P). We also treated successional stage and functional group identity as fixed effects. We started from full models, which included multiple explanatory variables (soil N:P, Net N mineralization, available P, total N, total P, stage and functional group) and multiple response variables (leaf N:P, stem N:P and root N:P). Final simplified models were retained based on reduction in AICs (Akaike Information Criterion, S7 Table), by removing non-significant terms. Each model was used to determine the order of predictors by its magnitude of influence on each of the outcome variable. We used linear Mixed Effects analysis for our final model where successional stages, functional group identity, soil N:P, available P and net N mineralization were treated as fixed effects, whereas the four primary succession sites were included as random effects. We addressed any potential significant effect of our fixed variables on plant N:P stoichiometry in different plant compartments. We also tested whether changes in plant N (%) and P (%) concentrations were related to changes in total soil N (%) and P (%) concentrations along the successions, using linear regression. We log transformed our response variables to make their values normally distributed. Mixed Effects Model was fitted using Restricted Maximum Likelihood (REML) algorithm to estimate the variance within the fixed-effect parameters and to provide the variance for the random-effect variables at once. Each of the stoichiometric response variables (i.e. leaf N:P; root N:P, stem N:P, soil N:P) was modelled as a function of the fixed effect parameters (i.e. succession stage, functional group, soil N:P and available P). We finally performed post hoc Tukey HSD tests to compare minimum distances between means of significant terms (N and P) in the Standard Least Squares Regression of succession types (dunes = S and glaciers = Gl) effect. Analyses were performed within the statistical software JMP versions 9 and 10 [40].

We also estimated the variation in nutrient concentrations and nutrient ratios among plant functional groups and across successional stages. We calculated the coefficient of variation (CV) from log_10_-transformed data using the same approach of Güsewell and Koerselman [11] and Wang and Moore [25]:

CVlog-normal=12[10(X¯+SD)-10(X¯-SD)]10X=10SD-10-SD2

Where $\bar{X}$ and SD are mean and standard deviation respectively. To test for differences among functional group means ($\bar{X}$), the nutrient concentrations and the stoichiometric ratios from all stages of the successions were averaged for each life-form. The SD was calculated with the means of each plant functional group and included in the formula. We calculated the coefficient of variation among soils in the same way considering differences (1) within the three successional stages, (2) among plant species occurring across the three stages, and (3) among different plant compartments for total N (%), total P (%) and N:P ratios (see Table 1).

**Table 1:** Coefficients of variation (%) of Nitrogen (N), Phosphorus (P) and N:P ratios.calculated for soils, plant species, plant compartments and plant functional groups (PFG) across our four primary successions.

Coefficients of variation (%)	N	P	N:P
Among soils across stages	134	7.3	145
Among plant species across stages	3.5	9.5	10.4
Among plant compartments	17.3	5.9	12
Among PFGs	45.9	19.9	31.6
PFGs across stages			
Grasses	6.4	7.4	4.9
Legumes	4.5	6.2	8.9
Forbs	3.7	15.2	18
PFGs between successions (Gl and S)			
Grasses			14.2
Legumes			26.1
Forbs			19.9

## Results

### Changes in soil and plant N:P ratios along successional stages

**Fig 1:**
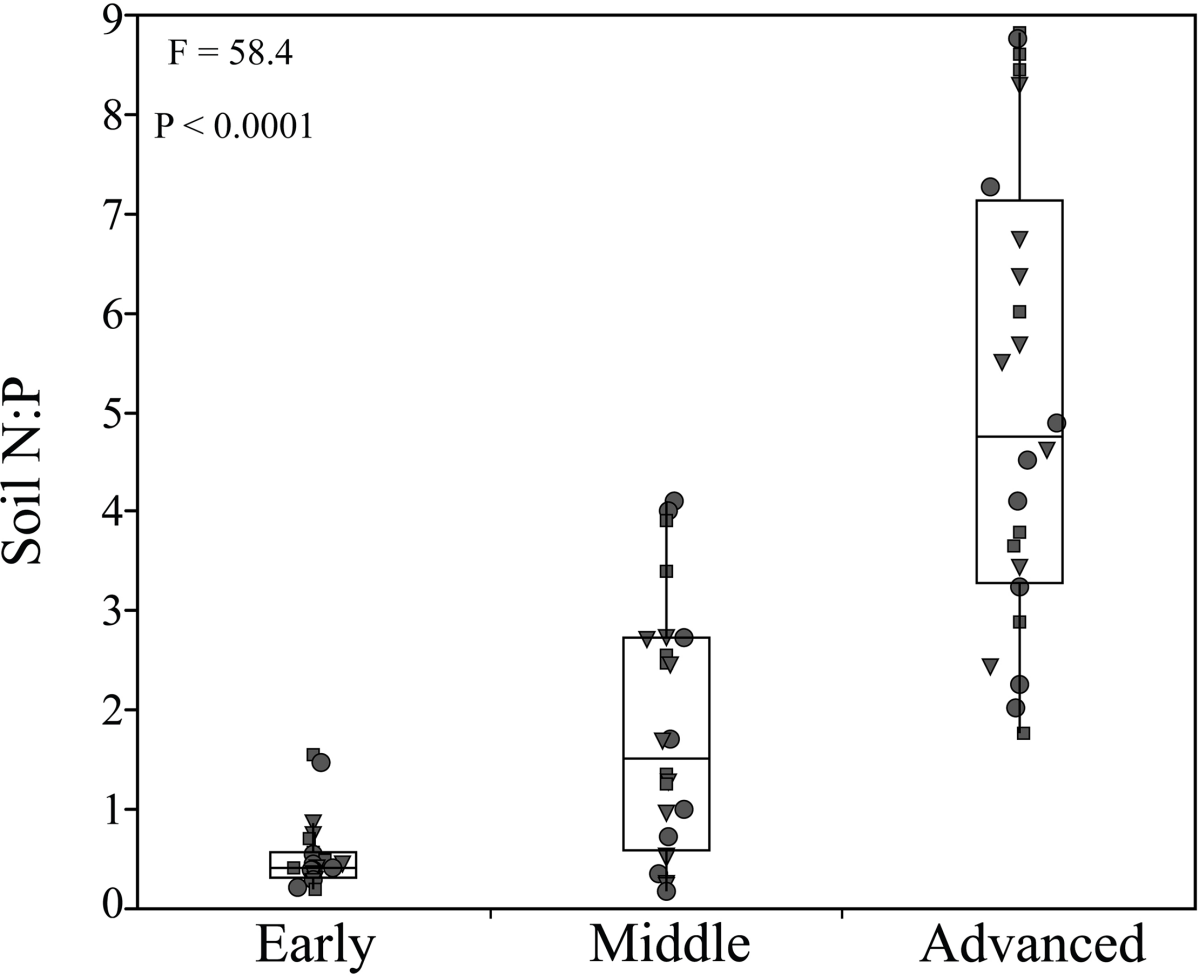
Variation in soil N:P ratios across increasingly older stages of the four primary successions. (Triangle = Grasses; Dots = Legumes; Squares = Forbs).

**Fig 2:**
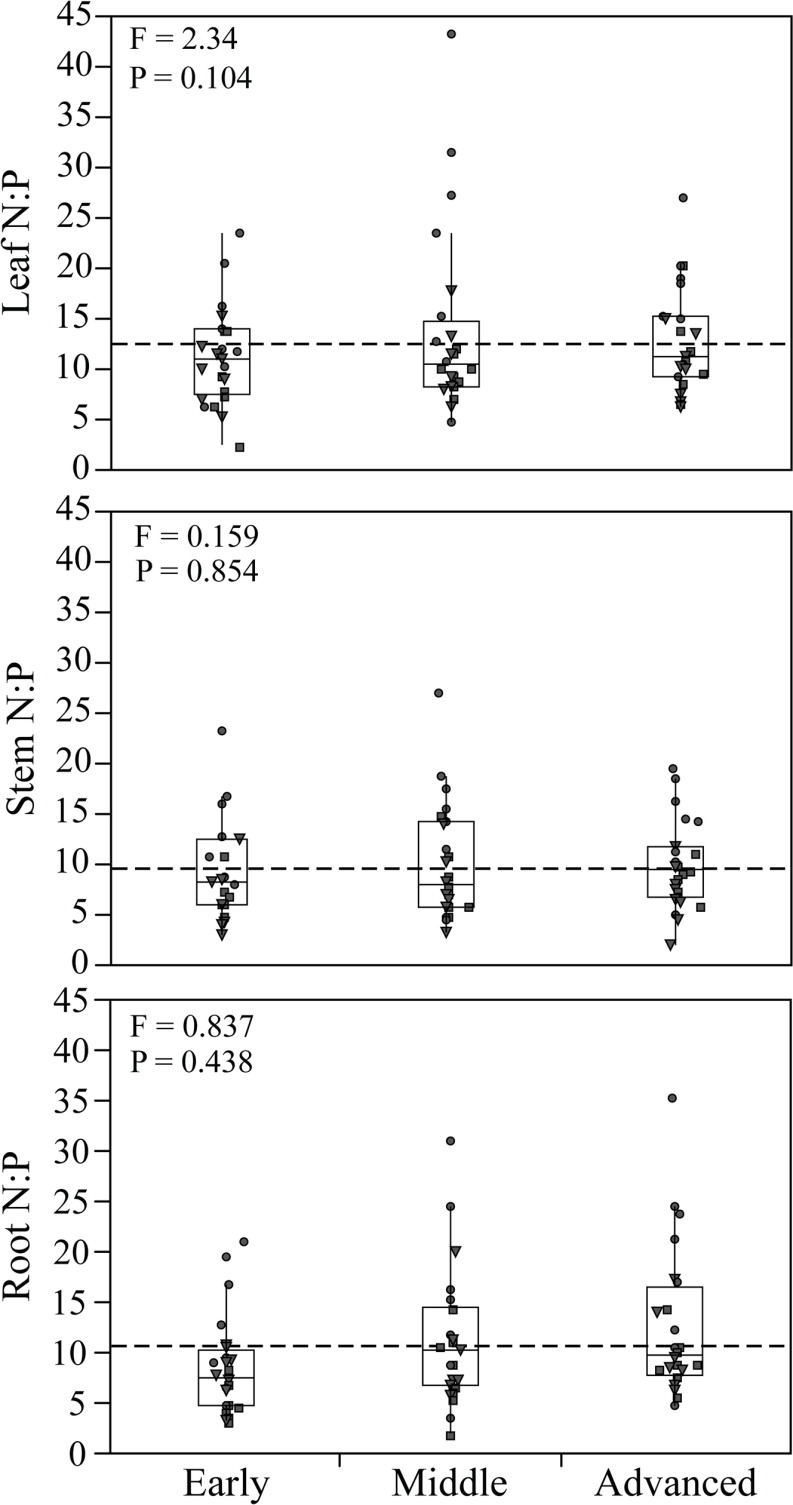
Variation in plant N:P ratios within different compartment (leaves, stems, roots) across the three successional stages (early, middle and advanced). Data points represent plant species belonging to different functional groups (Triangle = Grasses; Dots = Legumes; Squares = Forbs).

**Fig 3:**
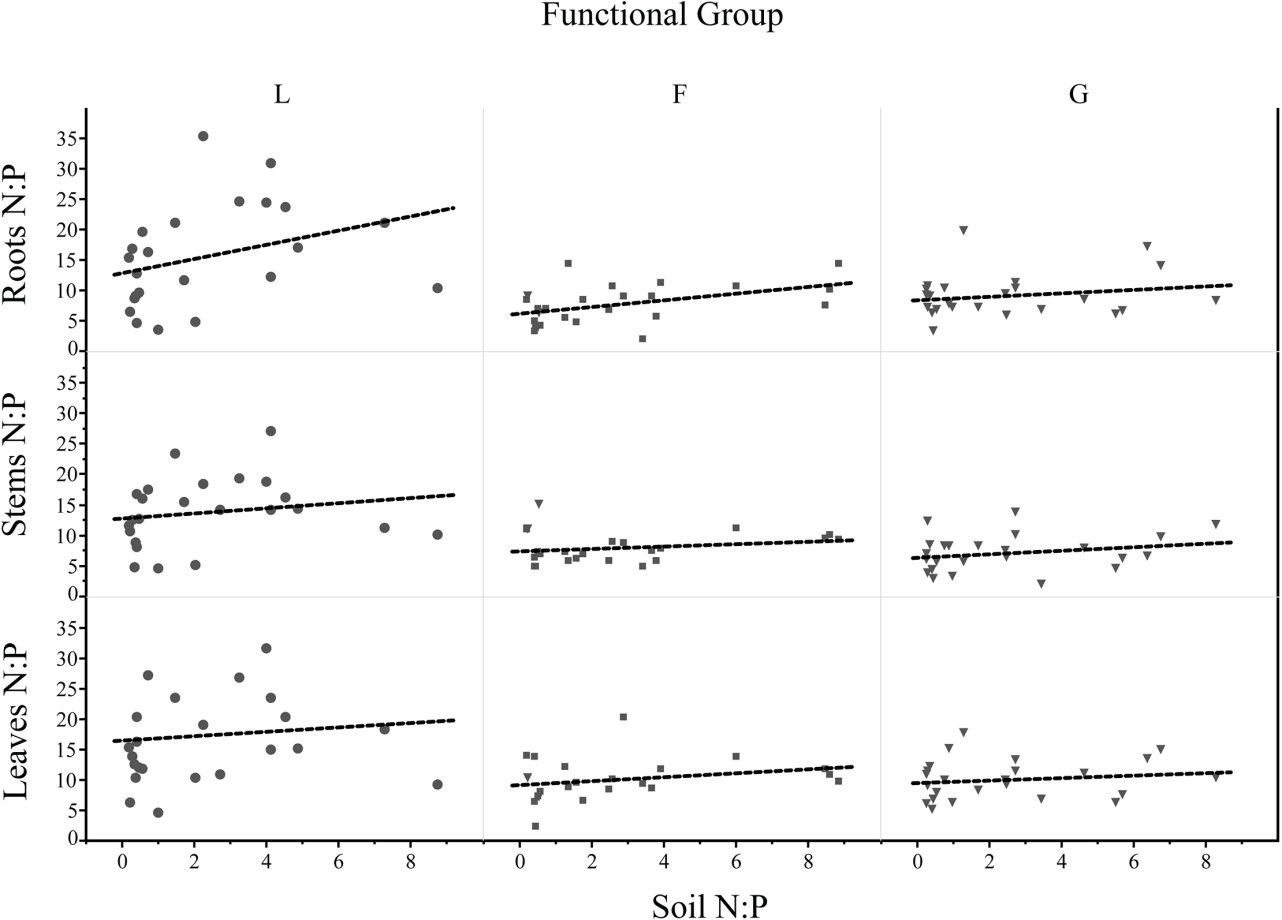
Relationships between soil N:P and plant N:P within each of three compartments (leaves, stems, roots) and across three functional groups. (Dots = L- legumes; Squares = F-forbs; Triangle = G-grasses).

We found that soil N:P ratios strongly increased along increasingly older stages of the four primary successions (*P* < 0.0001; Fig 1; Table 1). In contrast we could not find any predictable variation in plant N:P stoichiometry in any plant compartment (leaves, shoots, roots) across the same successional stages (Fig 2). Only roots showed a positive trend (although not significant) in their tissue N:P ratio along our primary successions (Fig 2), which was similar to changes in soil N:P ratios (Table 2). When we included in our full model multiple soil parameters (i.e. net soil N mineralization rates and available P) and categories (i.e. successional stage and functional group identity) as predictor variables (Table 3), we did not find any significant relationship between any of the soil parameters and plant N:P stoichiometry (see also Fig 3; S3 and S4 Figs).

**Table 2:** Means and Standard Deviations (SD) for total N (%), total P (%) and N:P ratios of different plant compartments and soils across successional stages.

	Leaf						Stem						Root						Soil					
	N		P		N:P		N		P		N:P		N		P		N:P		N		P		N:P	
	*Mean*	*SD*	*Mean*	*SD*	*Mean*	*SD*	*Mean*	*SD*	*Mean*	*SD*	*Mean*	*SD*	*Mean*	*SD*	*Mean*	*SD*	*Mean*	*SD*	*Mean*	*SD*	*Mean*	*SD*	*Mean*	*SD*
Early	1.63	0.80	0.15	0.06	11.2	4.82	1.37	0.58	0.16	0.05	9.22	4.80	1.24	0.62	0.16	0.09	8.67	4.79	0.03	0.02	0.06	0.04	0.52	0.35
Middle	1.79	0.96	0.17	0.16	13.7	9.16	1.29	0.61	0.15	0.09	10.1	5.71	1.34	0.73	0.15	0.11	11.1	6.74	0.07	0.04	0.06	0.03	1.80	1.27
Advanced	1.88	0.97	0.15	0.07	12.9	5.26	1.19	0.68	0.13	0.06	9.81	4.32	1.35	0.65	0.12	0.04	12.6	7.36	0.32	0.27	0.05	0.03	5.17	2.32

**Table 3:** Effects of soil parameters and functional group (FG) identity on N:P ratios of different plant compartments and entire plant samples. Note the significant effect of FGs when treated as single effect and also when nested within successional stages.

		Plant N:P			Leaf N:P			Stem N:P			Root N:P	
	*DF*	*F*	*P value*	*DF*	*F*	*P value*	*DF*	*F*	*P value*	*DF*	*F*	*P value*
*Stage*	2	1.27	0.29	2	1.18	0.313	2	1.7595	0.181	2	0.774	0.465
*FGs*	2	22.9	< 0.0001	2	12.5	< 0.0001	2	23.09	< 0.0001	2	15.06	< 0.0001
*Net N min*	1	0.537	0.475	1	0.434	0.528	1	2.79	0.116	1	0.0025	0.960
*AvP*	1	0.616	0.435	1	0.353	0.558	1	0.0999	0.753	1	1.11	0.296
*Soil N*:*P*	1	1.69	0.197	1	0.409	0.525	1	4.86	0.0312	1	0.503	0.481
*FGs[stage]*	6	7.52	< 0.0001	6	4.77	0.0005	6	7.34	< 0.0001	6	5.17	0.0003

**Fig 4:**
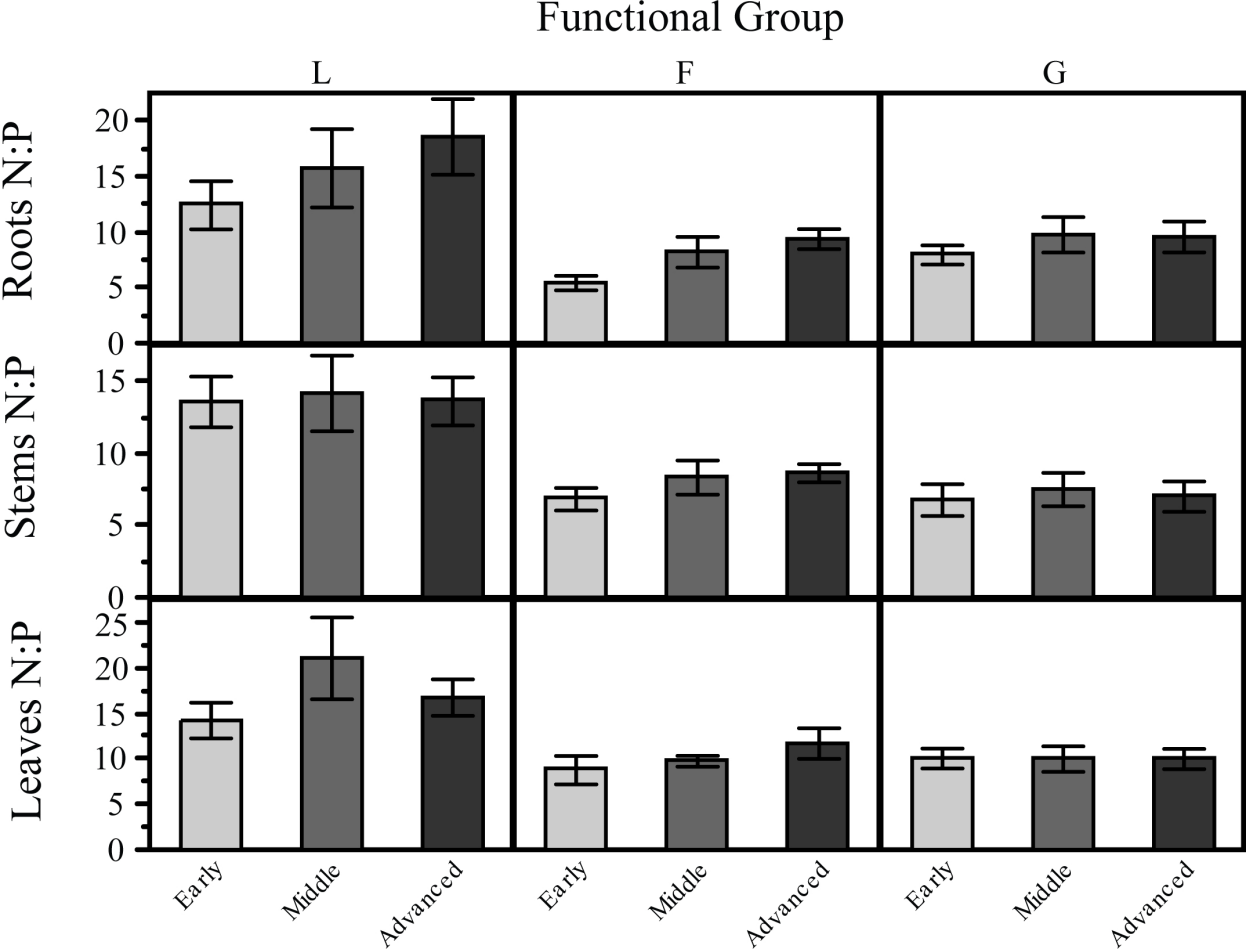
Dependence of plant N:P stoichiometry (leaves, stems and roots) on plant functional group (PFG) identity across successional stages. Legumes (L) show the highest N:P ratios compared with Graminoids (G) and Forbs (F).

**Fig 5:**
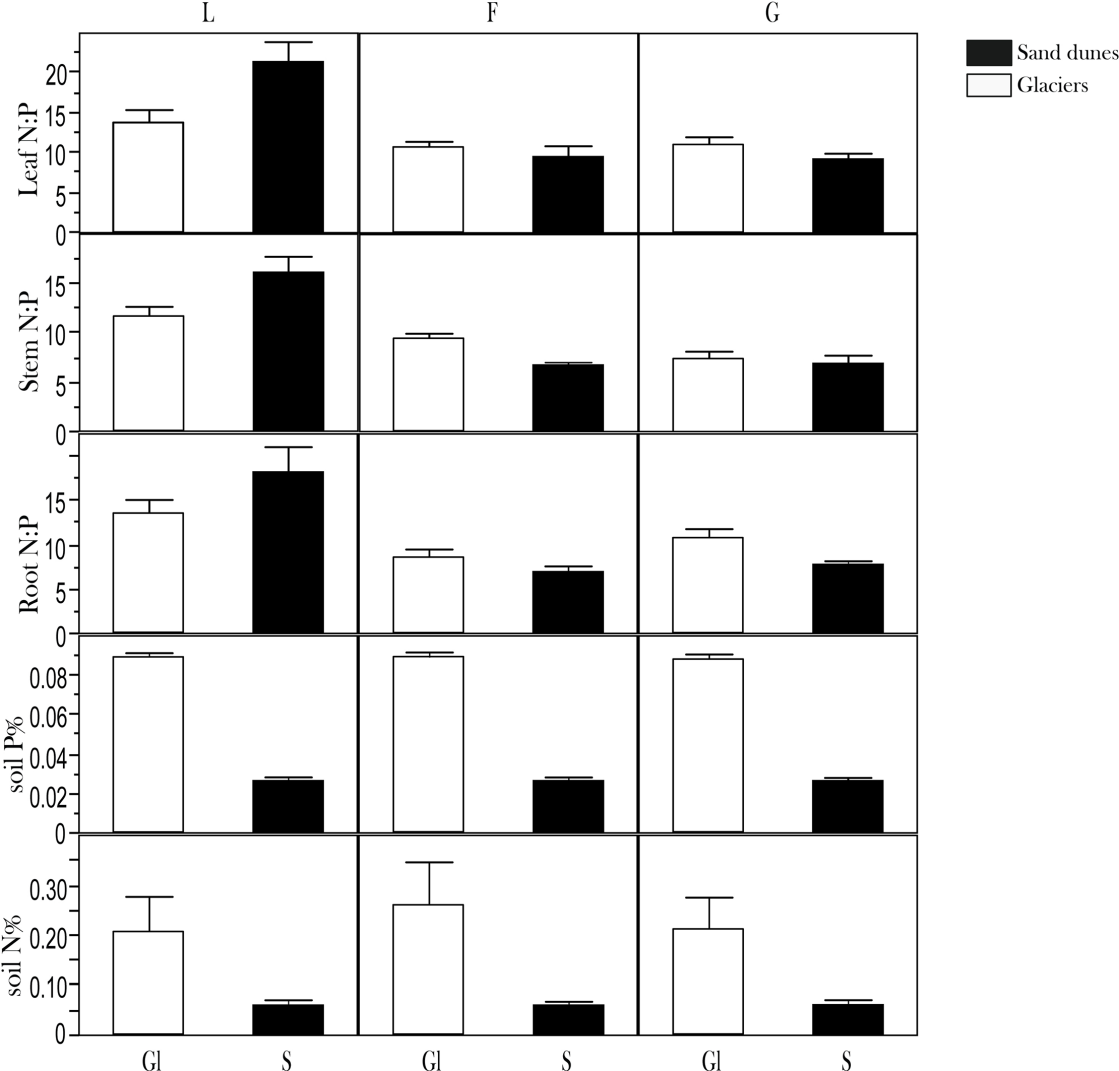
Patterns of plant nutrient stoichiometry and soil N% and P% concentrations between glacier (Gl) and sand dune (S) systems. Plant N:P ratios refer to: legumes (L), Graminoids (G) and Forbs (F). The difference in N (%) and P (%) between glaciers and dunes are reported also as t test value (N%: α = 0.050; t = 1.99; P = 0.0004; Glacier system Mean = 0.223: Dune System Mean = 0.058; P%: α = 0.050; t = 1.99; P = <0.0001; Glacier System Mean = 0.088; Dune System Mean = 0.026).

In our mixed model the only factor significantly influencing plant N:P stoichiometry was plant functional group identity (Fig 4; Table 3).To further test for potential effects of functional group identity on plant nutrient stoichiometry, we ran our analysis where plant functional group identity was nested within successional stage. We found that functional group identity was the most important variable affecting plant stoichiometry within each stage of the succession (Table 3). We also found that, within each plant functional group, variation in N:P ratios was low (coefficient of variation <18%) across successional stages and that grasses showed the lowest variability keeping their N:P ratios relatively constant across successional stages (Fig 4; Table 1). This despite the fact that variation in soil N:P ratios along the same successional stages was >145% (Table 1). We finally compared the relative effect of plant functional group identity with the “nature” of our primary successions (i.e. dunes vs. glaciers) on changes in plant N:P stoichiometry. Total soil N (%) and total soil P (%) were significantly higher at glaciers sites (Fig 5). The only functional group that responded to changes in the nature of the primary successions were legumes which had higher N:P ratios in leaves, roots and stems in the sand dune successions. Forbs and grasses show low and relatively ‘constant’ N:P ratios across both glacier and sand dune successions. The coefficient of variation of N:P ratios in different plant compartments across glacier and sand dune systems was low in general (Table 1).

### Changes in total soil and plant nutrient concentrations along successional stages

Changes in total soil N (%) and P (%) content also followed a clear pattern across successional stages whereby soil N (%) increased from early to advanced successional stages and soil P (%) showed an opposite trend (Table 2). Foliar and root N concentrations increased along the three successional stages (from early to advanced) reflecting the soil N gradient that we found along the same three successional stages (Table 2). We observed though an opposite trend for N (%) concentrations in plant stems, which decreased from early to advanced stages. Generally total P concentrations in soils and in plant tissues decreased along the three successional stages (Table 2).

## Discussion

Overall our results show that soil N:P ratios strongly increased along progressively older stages of the four primary successions, whereas plant N:P ratios did not. Thus we reject our first hypothesis that soil and plant N:P ratios are positively related along gradients of soil development. Changes in plant N:P ratios were not either related to changes in soil N and P availability (S3 and S4 Figs) but were rather ‘controlled’ by plant functional group identity. These results support our second hypothesis and agree with previous findings, which suggest that plant functional group identity can strongly influence N:P stoichiometry of different plant compartments [11, 25, 39, 41].

Previous studies suggest that the lack of correlation between plant and soil N:P stoichiometry might be due to high internal nutrient regulation by plants [11, 13–14]. We could not test this potential ‘plant regulation effect’ because individuals of only two plant species occurred across all successional stages (i.e. *Lotus creticus*, *Anthyllis vulneraria*). Our suggestion, however, is that an internal control of nutrient use and allocation might exist at least at the ‘plant functional group’ level because (1) legumes always showed significantly higher plant N:P ratios when compared to grasses and forbs, and (2) all plant functional groups had relatively constant N:P ratios across the three successional stages. In a similar alpine ecosystem Zhao et al. [41] also found that ‘plant growth form’ was more important than soil or climate in explaining changes in leaf C:N:P stoichiometry. Moreover, a recent study [42], which addressed plant and soil N:P stoichiometry along a chronosequence of volcanic substrates spanning 60,000 years, show similar findings that (1) foliar N:P ratio did not change along the chronosequence, (2) foliar N:P ratios were controlled by plant functional group identity (evergreen vs. deciduous), and (3) plant and soil N:P ratios were decoupled (i.e. not related) along the ecological succession.

In our natural plant communities N could enter the soil compartment through atmospheric N deposition, biological nitrogen fixation and the decomposition of organic matter in top-soils. We thus expect that N will initially accumulate faster in ‘legume-conditioned’ soils and then will increase in late development stages where more plant litter is returned to the soil [43–44]. Legumes are N-fixers thus produce their own N supply, which could partly explain legumes’ ability to maintain higher N:P ratios than the other plant functional groups. The fact that legumes, grasses and forbs were able to maintain their N:P ratios relatively constant across the soil development gradient, raises questions about what set of plant eco-physiological mechanisms are ultimately responsible for ‘controlling’ plant internal nutrient stoichiometry.

One potential explanation is that wild plants by stabilizing internal N and P tissue concentrations can better preserve cells from early senescence and avoid possible toxicity from excessive N and P concentrations in plant tissues [45–46]. In our soils, plant internal control on tissue N:P ratios may have developed to reduce nutrient losses [16]. It has been shown that internal stoichiometric balance of graminoids adapted to nutrient-poor soils depends on the reallocation of nutrients from shoots to roots and from old to young leaves [47–48]. These mechanisms may involve up- and down-regulating of N and P uptake, and the allocation and transport of these nutrients across compartments based on different physiological needs [49]. For instance amino acids that are not invested in shoot growth may be withdrawn from aboveground compartments and immobilized in roots [50]. Plant species growing on N-limited soils may have also developed different strategies to get access to several different N sources including inorganic and organic soil N forms, atmospheric N_2_, and N in precipitation [16].

Nutrient uptake and nutrient internal stoichiometric regulation could depend on competitive interactions among plant species and on the frequency of disturbance events such as drought, ice, fire and pathogens [3, 51–53]. Harsh and highly variable environments such as glacier and sand dune systems, host an array of stress tolerant plants, which may have developed an optimum of N and P use efficiency as a unique ecosystem response to extreme conditions [54]. These plants show high internal nutrient regulation, which is common in ecosystems where plants are co-limited by both N and P [7]. Foliar and root N:P ratios in our plants changed from 11 and 9 respectively in early successional stages to ≈13 in advanced successional stages, which according to previous studies should indicate a trend towards N and P co-limitation [13–14], and a dependency on soil nutrient availability, which however was not found in our study. Higher N:P ratios would suggest increasing P-limitation compared to N. It is however difficult to establish at which point P will become more limiting than N for plant growth and to what extent this will affect plant stoichiometry. In a long-term grassland experiment Fornara et al. [55] found that plant communities which received only N for almost 20 years (thus becoming P-limited) had an average root N:P ratio of 13.8 which is similar to the N:P ratio of our roots in advanced stages of the primary successions.

Recent nutrient fertilization experiments show that plant stoichiometry can greatly change depending on nutrients availability in soils [22, 55–56]. In our study we found that plant N:P stoichiometry was neither related to changes in soil N and P availability nor to changes in total soil N and P content. It could be that in their natural environment wild plants have developed particular nutrient allocation strategies (compared to plants under experimental conditions), which contribute to moderate the effects of external nutrient variability.

Plants adopt different strategies to optimize resource use and their N and P resorption abilities may change with soil age. For example, fast growing non-mycorrhizal plants and plants with arbuscular mycorrhizal associations are able to colonize N-limited young soils because they can access soluble inorganic and some soluble organic N sources from soils. On older soils, where P is lost via leaching and erosion, plants with cluster roots and the ability to exude carboxylates are advantaged in mining for P sources [57]. However, other non-mycorrhizal, carboxylate-exuding species that ‘mine’ P from the soil may benefit under P-poor soil conditions but net benefits will also depend on the interaction with other plant growth traits (e.g. N-fixation is associated with higher plant-P requirements; [57]).

Thus tissue N and P concentrations may not always reflect the availability of N and P in soils, because their availability is relative to the ability of plants in mobilizing and absorbing them [58–59]. Interspecific interactions (e.g. facilitation) between different groups of plants (i.e. legumes and grasses) could well explain the lack of plant-soil N:P relationships in our primary successions. For example, legume species on young N-limited soils are able to exude organic acids through their roots to improve their uptake of P from Al-, Ca- and Fe- bound minerals [60]. This represents another potential legume-induced mechanism to increase soil P mobility, which then benefits P-uptake by other plants, particularly by grass species [61].

Previous studies, which focused on much longer ecological successions (> 2 million-year-long), show strong effects of soil nutrient availability on plant nutrient-use efficiency, which are consistent with a shift from N to P limitation [62, 63]. These studies report a clear increase in the functional diversity of belowground traits related to nutrient acquisition during ecosystem development. The existence of multiple N- and P-acquisition strategies together with high spatial variability in N and P availability in soils might explain why plant and soil N:P stoichiometry may not be necessarily related along primary ecological successions.

## Conclusions

Overall we found that soil and plant N:P stoichiometry are poorly related along primary ecological successions. We suggest that evolutionary and functional traits related to resource uptake and use might exert a primary ‘control’ on wild plant nutrient stoichiometry. Thus variation in plant tissue N:P ratios may not be a good predictor of N and P limitation along gradients of soil development in primary successions, which have not reached yet a retrogressive stage. By comparing different types of succession (i.e. dunes and glaciers), we also found that legumes had higher N:P ratios in sand dune systems suggesting that legume growth might be more P-limited in these systems. Finally, grasses and forbs showed more constancy in their N:P ratios both across sand dune and glacier successions. Further studies could investigate how wild plant species may develop different nutrient-acquisition strategies and/or regulate internal nutrient use to ‘maintain’ plant tissue stoichiometry stable despite changes in the chemistry of the surrounding environment. Finally, we suggest that combining a nutrient stoichiometric approach with the measurement of key plant functional traits across temporal and spatial scales can greatly improve our mechanistic understanding of how plant species are distributed along environmental gradients.

## Supporting information

S1 Data (XLSX)

S1 Fig Distribution of the four soil chronosequences: Umbra Forest Natural Reserve, Northern Ireland (top left); Belvedere Glacier, Italy (top right); Delle Locce Glacier, Italy (bottom right), and Parque Natural do Sudoeste Alentejano e Costa Vicentina, Portugal (bottom left). (PDF)

S2 Fig Study design: 4 block sites, 3 successional stages, 6 plant species collected within each stage (i.e. 2 legume, 2 grass, 2 forb species). (PDF)

S3 Fig Lack of significant relationships between plant N:P ratios and net soil nitrogen mineralization rates. (PDF)

S4 Fig Lack of significant relationships between plant N:P ratios and available soil P. (PDF)

S5 Fig N:P ratios of all plant species belonging to the three functional groups (L = legumes; F = Forbs; G = Grasses) across the three stages of the successions. For each functional group plant N:P ratio is the same across the stages.
(PDF)

S1 Table Details of the four primary successions. The age of each successional stage has been estimated from literature: *a* Moura *et al*. 2006; Dias *et al*. 2000; Clark and Rendell 2006; *b* Wilson et el. 2004; Wilson P. 1987; *c* Caccianiga and Andreis 2004; Monterin 1919; Sacco 1938; Smiraglia 1992; Korner 2003.
(PDF)

S2 Table Data of frequency of plant species at Belvedere Glacier succession obtained from transects repeated 6 times (A-F) in each stage of the plant community observed. The column of total individuals for each species reported for each stage was used to estimate qualitative plant species abundance.
(PDF)

S3 Table Data of frequency of plant species at Locce Glacier succession obtained from transects repeated 6 times (A-F) in each stage of the plant community observed. The column of total individuals for each species reported for each stage was used to estimate qualitative plant species abundance.
(PDF)

S4 Table Data of frequency of plant species at Carrapateira Dune succession obtained from transects repeated 6 times (A-F) in each stage of the plant community observed. The column of total individuals for each species reported for each stage was used to estimate qualitative plant species abundance.
(PDF)

S5 Table Data of frequency of plant species at Umbra Dune succession obtained from transects repeated 6 times (A-F) in each stage of the plant community observed. The column of total individuals for each species reported for each stage was used to estimate qualitative plant species abundance.
(PDF)

S6 Table List of the plant species collected across the four primary ecological successions and separated between the three broad plant functional groups. (PDF)

S7 Table AICs value for the selection of best-fitted model. For each of the Y variables (i.e. leaf N:P, stem N:P and root N:P) a series of models have been provided through stepwise regression analysis in JMP and best fitted models were selected for the smallest AICs value.
(PDF)
