# Supplementary figures and images for: Plant and soil nutrient stoichiometry along primary ecological successions: Is there any link?
Source: PLoS One. 2017 Aug 7;12(8):e0182569. doi: 10.1371/journal.pone.0182569 (PMC5546702; doi:10.1371/journal.pone.0182569)

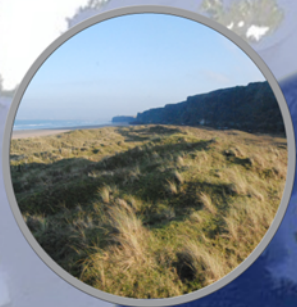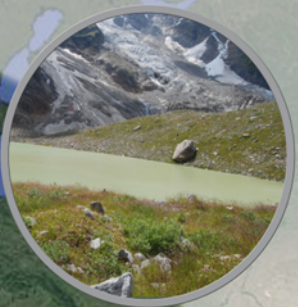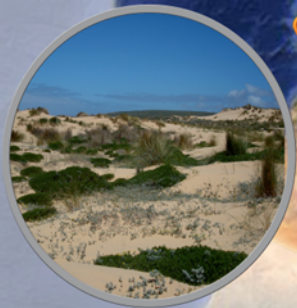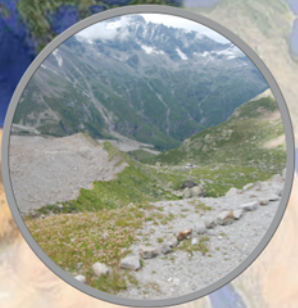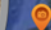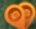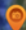

Supplement: S1 Fig — (PDF) [file pone.0182569.s002.pdf]

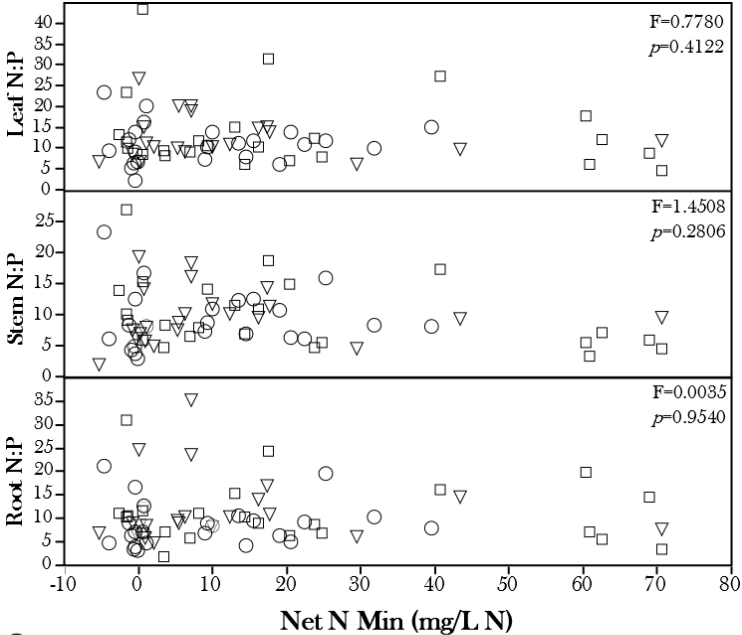

○ Early Stage

□ Middle Stage

▽ Advanced Stage

Supplement: S3 Fig — (PDF) [file pone.0182569.s004.pdf]

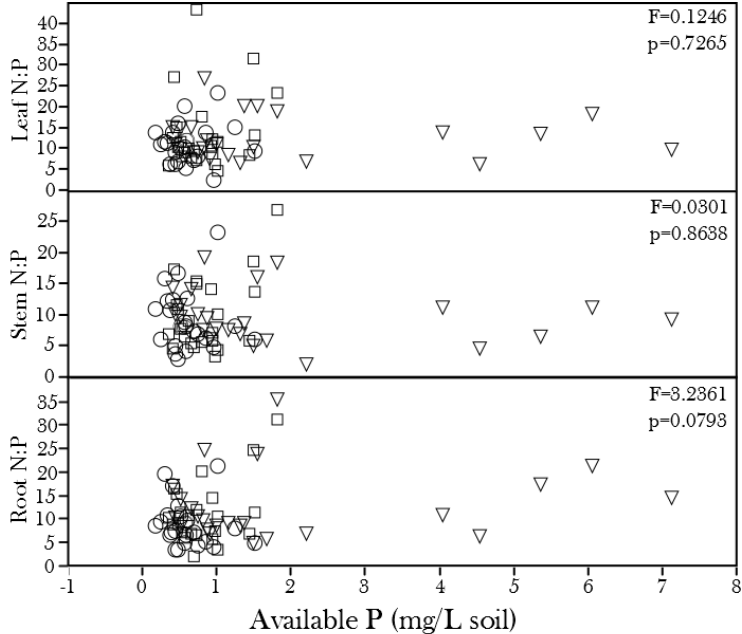

- Early Stage
- Middle Stage
- ▽ Advanced Stage

Supplement: S4 Fig — (PDF) [file pone.0182569.s005.pdf]

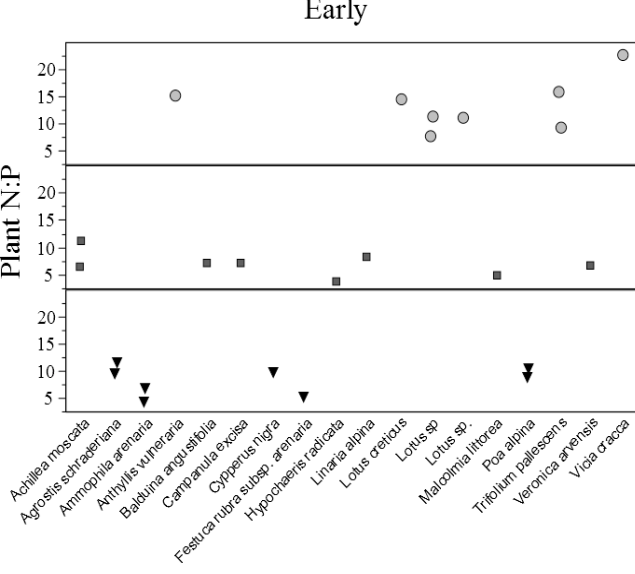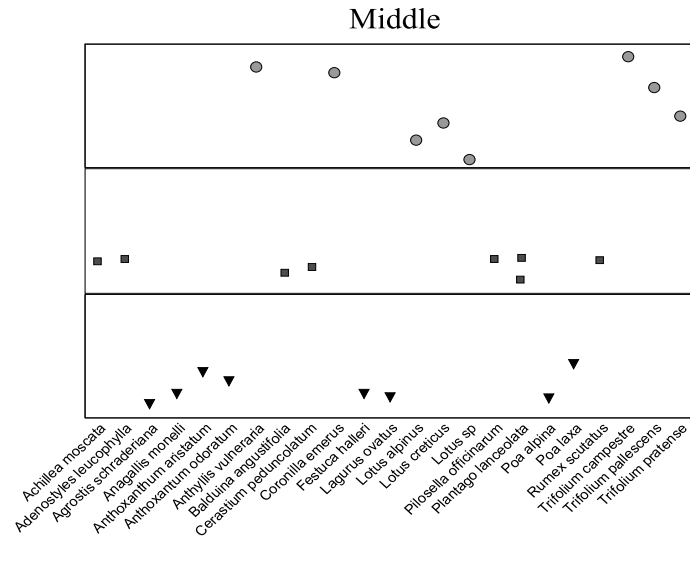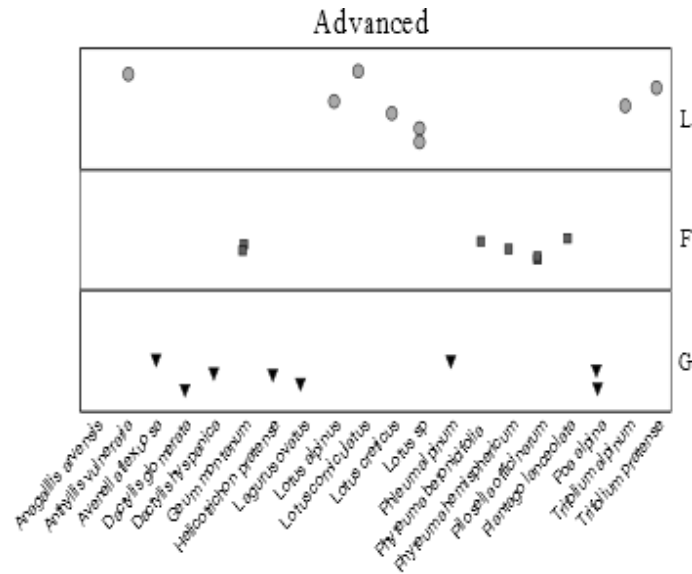

Supplement: S5 Fig — For each functional group plant N:P ratio is the same across the stages. (PDF) [file pone.0182569.s006.pdf]
