# Supplementary material for: Plant and soil nutrient stoichiometry along primary ecological successions: Is there any link?
Source: PLoS One. 2017 Aug 7;12(8):e0182569. doi: 10.1371/journal.pone.0182569 (PMC5546702; doi:10.1371/journal.pone.0182569)
Supplement: S2 Fig — (PDF) [file pone.0182569.s003.pdf]

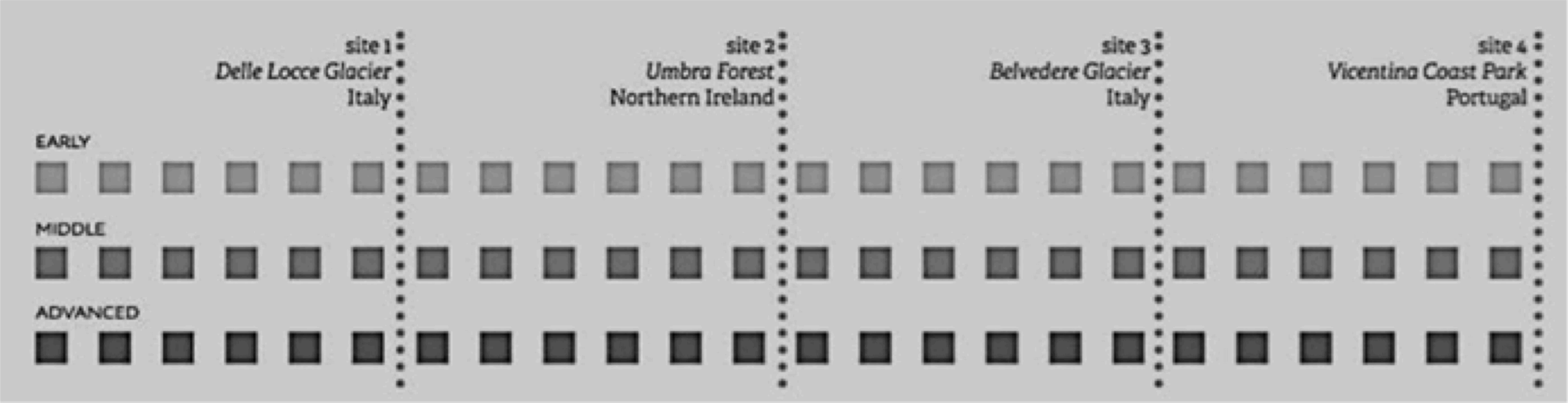
site 1
Delle Locce Glacier
Italy
site 2
Umbra Forest
Northern Ireland
site 3
Belvedere Glacier
Italy
site 4
Vicentina Coast Park
Portugal
EARLY
MIDDLE
ADVANCED
