# Supplementary material for: Plant and soil nutrient stoichiometry along primary ecological successions: Is there any link?
Source: PLoS One. 2017 Aug 7;12(8):e0182569. doi: 10.1371/journal.pone.0182569 (PMC5546702; doi:10.1371/journal.pone.0182569)
Supplement: S1 Table — The age of each successional stage has been estimated from literature: a Moura et al. 2006; Dias et al. 2000; Clark and Rendell 2006; b Wilson et el. 2004; Wilson P. 1987; c Caccianiga and Andreis 2004; Monterin 1919; Sacco 1938; Smiraglia 1992; Korner 2003. (PDF) [file pone.0182569.s007.pdf]

Study sites	Annual Mean Temperature (°C)	Annual Mean Precipitation (mm)	Soil pH	Succession Age (years)		
				Early	Middle	Advanced
Carrapateira Dunes[a]	15.9	563	8.7	<50	200-600	>1000
Umbra Dunes[b]	8.5	1065	8.6	<50	200-600	>1000
Belvedere Glacier[c]	5	1695	5.5	<50	200-600	>1000
Locce Glacier[c]	5	1695	5.2	<50	200-600	>1000

References

Caccianiga M, Andreis C. (2004) Pioneer herbaceous vegetation on glacier forelands in the Italian Alps. *Phytocoenologia* 34: 55-89.

Clark ML and Rendell HM. (2006) Effects of storminess, sand supply and the North Atlantic Oscillation on sand invasion and coastal dune accretion in western Portugal. *Holocene* 16: 341-355.

Dias JMA, Boski T, Rodrigues A, Magalhães F. (2000) Coast line evolution in Portugal since the Last Glacial Maximum until present-a synthesis. *Marine Geology* 170: 177-186.

Körner C. (2003) *Alpine Plant Life: functional plant ecology of high mountain ecosystems.* 2nd ed., XI, 349 p. Springer Ed.

Monterin U. (1919) Introduzione allo studio del ghiacciaio italiano del Monte Rosa. *Bollettino del Comitato Glaciologico. Italiano* 3: 105-164.

Moura D, Veiga-Pires C, Albardeiro L, Boski T, Rodrigues AL, Tareco H. (2006) Holocene sea level fluctuations and coastal evolution in the Central Algarve (Southern Portugal). *Marine Geology,* 237, 127-147.

Sacco F. (1938) Il glacialismo piemontese. *L'Universo, Torino*. 19: 217-352.

Smiraglia C. (1992) *Guida ai Ghiacciai e alla Glaciologia*. Zanichelli, Bologna.

Wilson P, McGourty J, Bateman MD. (2004) Mid-to late-Holocene coastal dune event stratigraphy for the north coast of Northern Ireland. *Holocene* 14: 406-416.

Wison P. (1987) Soil formation on coastal beach and dune sands at Magilligan Point Nature Reserve, Co. Londonderry. *Irish Geography* 20: 43-50.
