# Supplementary material for: Plant and soil nutrient stoichiometry along primary ecological successions: Is there any link?
Source: PLoS One. 2017 Aug 7;12(8):e0182569. doi: 10.1371/journal.pone.0182569 (PMC5546702; doi:10.1371/journal.pone.0182569)
Supplement: S6 Table — (PDF) [file pone.0182569.s012.pdf]

Legume Species	Graminoid Species	Forb Species
**Early Stage**<br><br>*Lotus creticus#*<br><br>*Lotus sp.*<br><br>*Anthyllis vulneraria#*<br><br>*Vicia cracca*<br><br>*Trifolium pallescens**<br><br>*Lotus alpinus**	**Early Stage**<br><br>*Ammophila arenaria**<br><br>*Cypperus nigra*<br><br>*Festuca rubia subsp. arenaria*<br><br>*Poa alpina**<br><br>*Agrostis schraderiana**	**Early Stage**<br><br>*Malcolmia littorea*<br><br>*Balduina angustifolia*<br><br>*Hypochaeris radicata*<br><br>*Veronica arvensis*<br><br>*Achillea moscata**<br><br>*Linaria alpina*<br><br>*Campanula excisa*
**Middle Stage**<br><br>*Lotus creticus#*<br><br>*Coronilla emerus*<br><br>*Anthyllis vulneraria#*<br><br>*Trifolium campestre*<br><br>*Trifolium pallescens*<br><br>*Lotus alpinus#*<br><br>*Trifolium pratense*	**Middle Stage**<br><br>*Lagurus ovatus#*<br><br>*Anagallis monelli*<br><br>*Anthoxantum odoratum*<br><br>*Anthoxanthum aristatum*<br><br>*Agrostis schraderiana#*<br><br>*Poa laxa*<br><br>*Poa alpina#*<br><br>*Festuca halleri*	**Middle Stage**<br><br>*Balduina angustifolia*<br><br>*Plantago lanceolata**<br><br>*Pilosella officinarum#*<br><br>*Cerastium peduncolatum*<br><br>*Rumex scutatus*<br><br>*Achillea moscata#*<br><br>*Adenostyles leucophylla*

Advanced Stage	Advanced Stage	Advanced Stage
Lotus creticus#	*Dactylis hyspanica*	*Pilosella officinarum**
Anthyllis vulneraria#	*Lagurus ovatus#*	*Anagalllis arvensis*
*Lotus corniculatus**	*Dactylis glomerata*	*Plantago lanceolata#*
Lotus alpinus#	*Helicotrichon pratense*	*Geum montanum**
Trifolium pratense	*Poa alpina**	*Phyteuma betonicifolia*
*Trifolium alpinum**	*Phleum alpinum*	*Phyteuma hemisphericum*
	Avenella flexuosa	

* Species found in the same stage (Early, Middle or Advanced) of two different successions (e.g. *Trifolium alpinum* was found in the advanced stage of both Belvedere and Locce Glaciers).

# Species found in two different successional stages of the same succession (e.g. *Plantago lanceolata* was found in Middle and Advanced stages of the Umbra sand dune system).
