# Supplementary material for: Plant and soil nutrient stoichiometry along primary ecological successions: Is there any link?
Source: PLoS One. 2017 Aug 7;12(8):e0182569. doi: 10.1371/journal.pone.0182569 (PMC5546702; doi:10.1371/journal.pone.0182569)
Supplement: S7 Table — For each of the Y variables (i.e. leaf N:P, stem N:P and root N:P) a series of models have been provided through stepwise regression analysis in JMP and best fitted models were selected for the smallest AICs value. (PDF) [file pone.0182569.s013.pdf]

Model	*N variables*	*R Square*	*RMSE*	*AICc*
Leaf N:P				
M1	1	0.2696	5.7943	448.948
**M2**	**2**	**0.2931**	**5.7427**	**448.912**
M3	3	0.3011	5.7532	450.437
M4	4	0.3121	5.7517	451.727
M5	5	0.3163	5.7787	453.771
M6	6	0.3174	5.8195	456.207
M7	7	0.3184	5.8620	458.745
Stem N:P				
M1	1	0.3675	3.9429	400.631
M2	2	0.3902	3.8998	400.282
**M3**	**3**	**0.4191**	**3.8346**	**399.151**
M4	4	0.4441	3.7794	399.417
M5	5	0.4539	3.7746	399.617
M6	6	0.4571	3.7929	401.745
M7	7	0.4602	3.8118	403.964
Root N:P				
M1	1	0.2715	5.5895	450.184
M2	2	0.3365	5.3736	445.803
**M3**	**3**	**0.3666**	**5.2894**	**444.826**

M4	4	0.3766	5.2868	446.078
M5	5	0.3792	5.3162	448.246
M6	6	0.3813	5.3486	450.553
M7	7	0.3823	5.3867	453.070

Leaf N:P

M1: FGs

M2: Stage; FGs

M3: Stage; FGs; %P

M4: Stage; FGs; %P; soil N:P

M5: Stage; FGs; AvP; %P; soil N:P

M6: Stage (early; middle; advanced); Stage (middle; advanced); FGs; AvP; %P; soil N:P

M7: Stage (e; m; a), Stage (m; a); FGs; %N; AvP; %P; soil N:P

Stem N:P

M1: FGs

M2: FGs; soil N:P

M3: FGs, Net N min; soil N:P

M4: Stage (m; a); FGs; Net N min; soil N:P

M5: Stage (m; a); FGs; Net N min; %P; soil N:P

M6: Stage (e; m; a); Stage (m; a); FGs; net N min; %P; soil N:P

M7: Stage (e; m; a); Stage (m; a); FGs; Net N min; %N; %P; soil N:P

Root N:P

M1: FGs

M2: FGs; AvP

M3: Stage (e, m, a); FGs; AvP

M4: Stage (e, m, a); FGs (G, L, F); FGs (G, F); AvP

M5: Stage (e, m, a); FGs (G, L, F); FGs (G, F); AvP; soil N:P

M6: Stage (e, m, a); Stage (m, a); FGs; AvP; soil N:P

M7: Stage (e, m, a); FGs (G, L, F); FGs (G, F); %N; AvP; %P; soil N:P
